# *Pseudomonas aeruginosa* quorum sensing inhibition by clinical isolate *Delftia tsuruhatensis* 11304: involvement of *N*-octadecanoylhomoserine lactones

**DOI:** 10.1038/s41598-019-52955-3

**Published:** 2019-11-11

**Authors:** Milka Malešević, Flaviana Di Lorenzo, Brankica Filipić, Nemanja Stanisavljević, Katarina Novović, Lidija Senerovic, Natalija Polović, Antonio Molinaro, Milan Kojić, Branko Jovčić

**Affiliations:** 1 Institute of Molecular Genetics and Genetic Engineering, University of Belgrade, Belgrade, 11010 Serbia; 2 University of Napoli Federico II, Department of Chemical Sciences, Napoli, 80126 Italy; 3 Faculty of Pharmacy, University of Belgrade, Belgrade, 11221 Serbia; 4 Faculty of Chemistry, University of Belgrade, Belgrade, 11000 Serbia; 5 Faculty of Biology, University of Belgrade, Belgrade, 11000 Serbia

**Keywords:** Antimicrobials, Biofilms

## Abstract

*Pseudomonas aeruginosa* is one of the most common opportunistic pathogens that use quorum sensing (QS) system to regulate virulence factors expression and biofilm development. *Delftia* sp. 11304 was selected among 663 Gram-negative clinical isolates based on its QS inhibitory activity against *P. aeruginosa* MMA83 clinical isolate. Whole genome sequencing identified this isolate as *D. tsuruhatensis* and revealed genetic armamentarium of virulence factors and antibiotic resistance determinants. Ethyl acetate extract of *D. tsuruhatensis* 11304 culture supernatant (QSI extract) prevented biofilm formation of *P. aeruginosa* MMA83, but was unable to cause biofilm decomposition. QSI extract showed a synergistic effect in combination with meropenem and gentamycin, against *P. aeruginosa* MMA83. A dose-dependent reduction of the virulence factors: elastase, rhamnolipid and pyocyanin production by *P. aeruginosa* MMA83 and significant downregulation of *lasI*, *lasR*, *rhlI*, *rhlR*, *pqs* and *mvfR* expression were observed. Matrix-assisted Laser Desorption Ionization (MALDI) mass spectrometry of *D. tsuruhatensis* 11304 QSI extract revealed the presence of *N*-acyl homoserine lactones (AHL) with chain lengths of C12 to C18. The main ion peak was identified as *N*-octadecanoylhomoserine lactone (C_18_-HSL). Commercial C_18_-HSL (20 µM) reduced pyocyanin production as well as mRNA level of the *lasI* gene. A novel AHL species, dihydroxy-*N*-octadecanoylhomoserine lactone, was also described.

## Introduction

*Pseudomonas aeruginosa* is one of the most significant opportunistic pathogens causing nosocomial infections with high morbidity and mortality, predominantly among immunocompromised and intensive care unit patients^1–4^. Pathogenic potential can be attributed to *P. aeruginosa* genomic plasticity and versatility which result in extensive genetic armamentarium of virulence factors and antibiotic resistance determinants^5^. Current increase in number of health care-associated infections caused by multidrug- (MDR) or extensively drug-resistant (XDR) *P. aeruginosa* isolates, as well as their worldwide spread has been considered as worrisome due to limited therapeutic options^6^. The lack of efficient therapeutic options for infections caused by MDR/XDR *P. aeruginosa* is driving research towards alternative therapeutic approaches such as targeting social behaviors involved in pathogenesis as well as bacteriophages and vaccines^7,8^.

*P. aeruginosa* cells communicate through quorum sensing (QS) system, i.e. by synthesizing small signal molecules, which depending on the density of the population correlate the regulation of virulence factors expression, biofilm development, production of secondary metabolites and interaction with hosts^9–11^. *P. aeruginosa* employs three major interconnected QS systems that function independently and dependently involving *las*, *rhl*, *pqs* pathways as well as novel candidate *iqs* pathway regulated by several QS signal molecules^12^. *N*-acyl homoserine lactones (AHLs) are the best characterized QS signal molecules. Different AHLs possess a homoserine lactone ring with an attached fatty acyl side chain of 4 to 20 carbons^13,14^. Detection of AHLs occurs either directly by LuxR regulators within the cell, or by membrane-bound two-component histidine kinase-type proteins^15,16^. Interestingly, long-chain AHLs, C_10_-AHL, C_12_-AHL and C_14_-AHL, were reported to bind to CviR AHL receptor in widely used reporter system *Chromobacterium violaceum* CV026 with the same affinity as C_6_-AHL autoinducer, but they disrupt activity of CviR in terms of transcriptional activation^17,18^. Disruption of QS achieved by interference with QS signaling or interception of signal molecules is considered a key point for development of antibacterial and anti-disease strategies targeting pathogens like *P. aeruginosa* in medicine^19^. Indeed, interference with QS signaling by QS-inhibitors (QSI) or interception of signal molecules by quorum quenching enzymes (QQE) results in a reduction of virulence regulated by QS. It is considered that QSI is a natural mechanism first developed either by QS-emitting organisms for the recycling or clearing of their own QS signals or by QSI organisms in the context of a competitive relationship with QS-signal-emitting organisms. Thus, bacteria that share ecological niche with *P. aeruginosa* during infections could be considered as promising producers of novel QSI molecules. Examples of the activity of QSI molecules, showing a successful reduction of siderophores, proteases, rhamnolipids secretion, as well as inhibition of biofilm formation have been previously documented. While those studies were mainly focused on *P. aeruginosa* PAO1 and PA14 model strains^9,20,21^ a still limited number of studies reported effects of QSI molecules on *P. aeruginosa* clinical isolates, especially MDR or XDR strains^22^. Diverse sources of QSI molecules that inhibit the virulence of *P. aeruginosa* have been described so far, both biogenic including plants, animals and bacteria^23–25^ or derived by chemical synthesis^26^.

*Delftia tsuruhatensis* strains have previously been investigated as plant growth-promoting rhizobacteria (PGPR) due to their production of siderophores which can mitigate iron limitation in soil^27^. However, recent findings pinpoint *D. tsuruhtensis* as an emerging pathogen associated with an increasing number of human infections^28–30^. It was previously published that *D. tsuruhatensis* could exhibit an anti-quorum sensing activity to *P. aeruginosa* quorum sensing systems, and authors identified a diisooctyl ester of 1,2 benzenedicarboxylic acid as an active compound^31^. Herein we analyzed inhibitory potential of *D. tsuruhatensis*11304 clinical isolate against QS systems of *P. aeruginosa* MDR clinical isolate and characterized molecule(s) underlying this phenomenon.

## Results

### Clinical isolate *Delftia* sp. 11304 produces QSI molecule(s)

**Figure 1:**
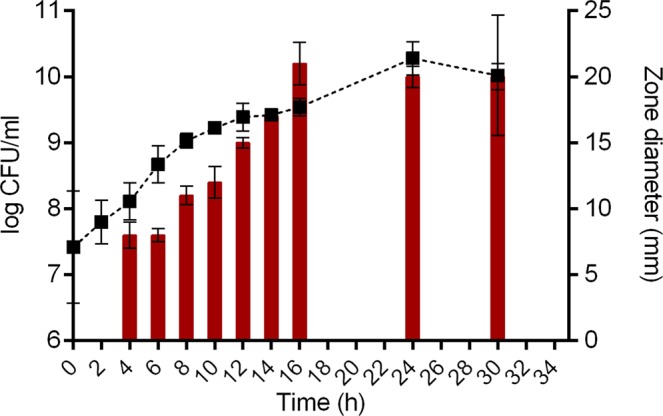
Growth phase dependence of QSI production by *Delftia* sp. 11304. Bacterial growth curve was determined by enumerating of colony forming units (CFU) at selected time points. Dependence of QSI activity from growth phase was determined at the same time points and expressed as numerical values obtained by measurement of inhibition zones of violacein production by *Chromobacterium violaceum* CV026.

Among 633 clinical isolates from a Laboratory collection screened for quorum sensing inhibitory (QSI) activity (Supplementary Table 1) 19 strains (belonging to five genera) were selected as positive (Supplementary Table 2). *Delftia* sp. 11304 was selected as one of the most promising candidate with QSI activity. Growth phase dependence of the *Delftia* sp. 11304 QSI phenotype was determined, as the QSI phenotype could not be detected before 4 hours of growth, while the peak of QSI activity was observed after16 hours (corresponding to the stationary phase). High production of QSI molecule(s) was retained after up to 30 hours of growth (Fig. 1). As a control QSI activity of previously characterized QSI molecules produced by *Delftia tsuruhatensis*: bis (2-ethylhexyl) phthalate and diisooctyl phthalate were tested in parallel. Neither of these compounds showed QSI activity at the used concentrations (ranging from 4 µg/ml to 1024 µg/ml) when tested in a colorimetric agar well diffusion assay using *Chromobacterium violaceum* CV026 as an indicator strain (Supplementary Fig. S1). Additionally, no measurable effects of bis (2-ethylhexyl) phthalate and diisooctyl phthalate neither on the viability of *C. violaceum* CV026 and *P. aeruginosa* MMA83 (Supplementary Table S3) nor to *P. aeruginosa* MMA83 biofilm production were detected (Supplementary Fig. S2).

### Whole genome sequencing

Genomic DNA of the strain *Delftia* sp. 11304 was sequenced using Illumina HiSeq. 2500 platform (MicrobesNG, University of Birmingham, United Kingdom). A total of 6,616,336 sequences were generated from the genome. Assembly of reads resulted in 199 contigs with the largest contig 337425 and an average GC content of 66.78%. The contig dataset was used to determine the functional analysis. According to the genomic sequence analysis *Delftia* sp. 11304 was identified as a *Delftia tsuruhatensis* species. A repertoire of different virulence factors and antibiotic resistance determinants were detected in *D. tsuruhatensis* 11304 genome (Supplementary Tables 4 and 5). Among the virulence factors presence of the genes encoding for different siderophores, pyoverdine, pyochelin and ornitobactin, as well as capsule and alginate could be pointed out. Additionally, within the *D. tsuruhatensis* 11304 resistome antibiotic resistance determinants such as genes encoding for efflux pumps of Resistance-Nodulation-Division (RND) superfamily (including: *mexF, mexY, mexB, mexD, mexI, mexA, smeB, smeE, amrB* and *mdtB*), beta-lactamases genes *bla*_OXA-258_ and *bla*_LRA-13,_ as well as spiramycin resistance gene were identified as the most significant.

### Stability of the QSI molecule(s)

**Figure 2:**
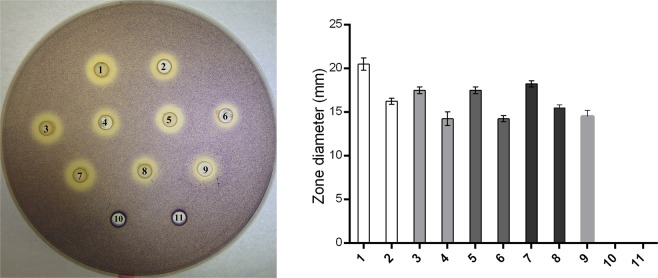
*D. tsuruhatensis* 11304 QSI activity was determined using colorimetric agar well diffusion assay with *Chromobacterium violaceum* CV026 as an indicator strain. (**a**) The activity of untreated control of *D. tsuruhatensis* 11304 overnight culture (1) and cell-free supernatant (2). The heat treatment at 100 °C for 30 min (3, 4) and 60 min (5, 6). *D. tsuruhatensis* 11304 overnight culture (3, 5) and cell-free supernatant (4, 6) retained QSI activity and shown to be thermostable. Proteinase K treatment did not affect QSI activity of *D. tsuruhatensis* 11304 overnight culture (7) and cell-free supernatant (8). Ethyl acetate extract of *D. tsuruhatensis* 11304 exhibits QSI activity (9). Proteinase K and LB growth medium with 0.5% DMSO (10, 11) are used as negative controls. (**b**) Quantitative data of these experiments obtained by measurement of inhibition zones of violacein production by *Chromobacterium violaceum* CV026.

In order to determine whether *D. tsuruhatensis* 11304 QSI activity was based on proteinaceous or non-proteinaceous molecule activity, thermostability and resistance to proteinase were tested. Results showed that both overnight culture and cell-free supernatant retained QSI activity after the heat and proteinase K treatments (Fig. 2). Obtained results suggested that the QSI activity of *D. tsuruhatensis* 11304 was based on non-enzymatic quorum sensing inactivation mechanism.

### Ethyl acetate *D. tsuruhatensis* 11304 extract exhibits QSI activity and inhibits biofilm formation of *P. aeruginosa* MMA83

Organic solvents with different polarity were used to optimize the extraction of the QSI molecule(s) from *D. tsuruhatensis* 11304 overnight culture supernatant. Only the ethyl acetate extract resuspended in DMSO was shown to be QSI active (Fig. 2), while methanol, chloroform and hexane extracts did not exhibit any QSI activity.

**Figure 3:**
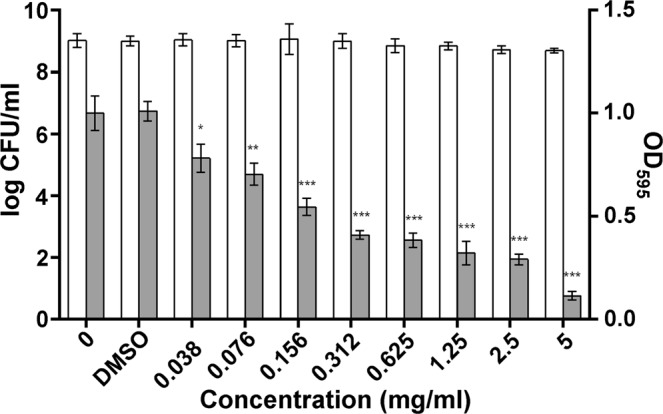
The effect of the *D. tsuruhatensis* 11304 QSI extract on *P. aeruginosa* MMA83 biofilm formation ability measured by crystal violet staining. The *D. tsuruhatensis* 11304 QSI extract inhibits biofilm formation in a dose-dependent manner. *P. aeruginosa* MMA83 cell growth is represented by white (logCFU/ml), and biofilm formation by gray bars (OD_595_). Numbers on x-axis represent two-fold serial dilutions of the *D. tsuruhatensis* 11304 QSI extract with the unit of measure being mg/ml. DMSO – bacterial culture with 0.5% v/v of DMSO that is the highest concentration of DMSO in experiment (used when 5 mg/ml of QSI extract was added). Data analysis by Student’s *t*- test demonstrates significant difference (*p < 0.05, **p < 0.01, ***p < 0.001) between the treated biofilms and the control (DMSO treated control). No significant difference in the cell growth between treated and untreated controls was observed.

To exclude that the QSI effect of extract was a result of a bactericidal effect on the *P. aeruginosa* MMA83, we evaluated the antimicrobial activity of the QSI extract by microdilution method. As shown in Fig. 3, *D. tsuruhatensis* 11304 QSI ethyl acetate extract had no statistically significant effect on the *P. aeruginosa* MMA83 growth.

Additionally, we have demonstrated that the inhibition of *P. aeruginosa* MMA83 biofilm formation by *D. tsuruhatensis* 11304 was dose-dependent using crystal violet staining of biofilm biomass (Fig. 3). Indeed, biofilm formation decreased by 88.5% in the presence of 5 mg/ml QSI extract comparing to the control (***p < 0.001). About 50% inhibition of biofilm formation was observed with a 0.156 mg/ml QSI extract (***p < 0.001). The lowest tested concentration of QSI extract (0.038 mg/ml) showed decreasing activity of 21.9% in regard to positive control (*p < 0.05). Treatment of *P. aeruginosa* MMA83 with the highest dose of DMSO used in experiment (0.5% v/v) had no statistically significant impact on bacterial growth and biofilm formation.

### *D*. *tsuruhatensis* 11304 QSI extract prevents biofilm formation of *P*. *aeruginosa* MMA83, but it is unable to induce biofilm decomposition

**Figure 4:**
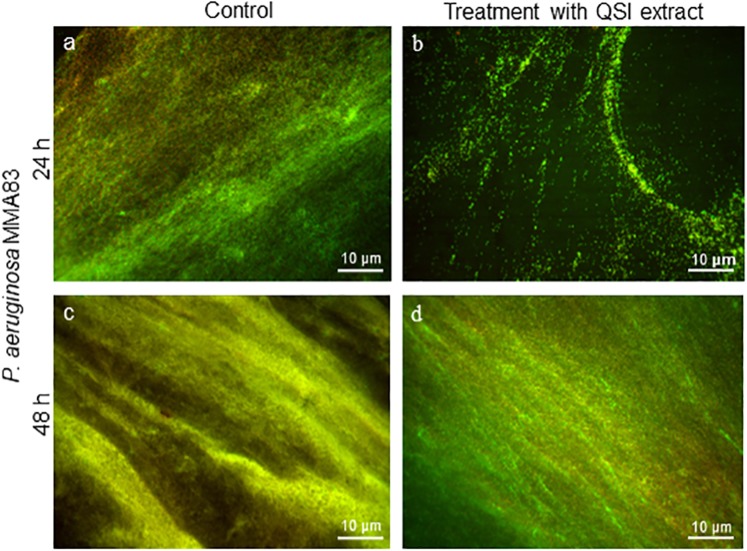
The effect of the *D. tsuruhatensis* 11304 QSI extract on *P. aeruginosa* MMA83 biofilm formation (**a**,**b**) and decomposition of preformed biofilms (**c**,**d**), captured with fluorescence microscopy. *P. aeruginosa* MMA83 was coincubated for 24 hours with 5 mg/ml *D. tsuruhatensis* 11304 QSI extract in order to visualize its effects on biofilm forming ability. The effect of the *D. tsuruhatensis* 11304 QSI extract on decomposition of 24 hours old preformed biofilm was evaluated after 24 hours of treatment with QSI extract. Fluorescent dyes SYTO9 (green) and PI (red) were used for the visualization of biofilms. Scale bar represents 10 µm.

Fluorescence microscopy has been used to visualize the effect of the QSI extract on *P. aeruginosa* MMA83 biofilm formation as well as on the decomposition of preformed biofilm. The captured images showed that *P. aeruginosa* MMA83 that grew in the absence of *D. tsuruhatensis* 11304 QSI extract formed a compact biofilm. In the presence of *D. tsuruhatensis* 11304 QSI extract biofilm was highly dispersed, with a clear decrease in the surface coverage and density of bacteria. Indeed, as shown in Fig. 4, minimal attachment of *P. aeruginosa* MMA83 cells could be observed in the treatment with a 5 mg/ml QSI extract (after 24 hours of incubation) (Fig. 4b).

Interestingly, co-incubation of preformed *P. aeruginosa* MMA83 biofilm with the QSI extract (5 mg/ml) did not result in decomposition of the biofilm (Fig. 4d), although the biofilm architecture was less consistent and slightly different compared to the positive control (Fig. 4c), there were still visible living bacterial cells resembling the architecture observed in the positive control.

### *D. tsuruhatensis* 11304 QSI extract shows synergistic effect with antibiotics

In order to investigate the clinical relevance of the QSI extract and its effectiveness in combination with clinically used drugs, the checkerboard method was assessed. Results indicate that the test strain *P. aeruginosa* MMA83 was susceptible to meropenem and gentamycin at very high used concentrations (MIC values 0.512 mg/ml and 4.096 mg/ml, respectively). MIC value of the *D. tsuruhatensis* QSI extract was 20 mg/ml. However, their combined application with the QSI extract showed a synergistic outcome for meropenem (∑FIC = 0.125) and for gentamycin (∑FIC = 0.047) (Table 1). DMSO (MIC value 145.45 mg/ml) had indifferent outcome for both of used antibiotics.

**Table 1:** The checkerboard method representing the effect of antimicrobials against *P. aeruginosa* MMA83.

Antimicrobials	Pseudomonas aeruginosa MMA83				
	MIC of each antimicrobial (mg/ml)		FIC	∑FIC	Outcome
	Alone	Combination			
QSI extract	20	1.25	0.0625	0.125	synergistic
Meropenem	0.512	0.032	0.0625		
DMSO	145.45	145.45	1	3	indifferent
Meropenem	0.512	1.024	2		
QSI extract	20	0.625	0.03125	0.047	synergistic
Gentamycin	4.096	0.064	0.0156		
DMSO	145.45	145.45	1	2	indifferent
Gentamycin	4.096	4.096	1		

FIC – fractional inhibitory concentration, FIC = MIC combination/MIC alone.

∑FIC – sum of two FICs, ∑FIC = FIC of antibiotic + FIC of QSI extract or DMSO.

### *D*. *tsuruhatensis* 11304 QSI extract inhibits the virulence factors production of *P*. *aeruginosa* MMA83

**Figure 5:**
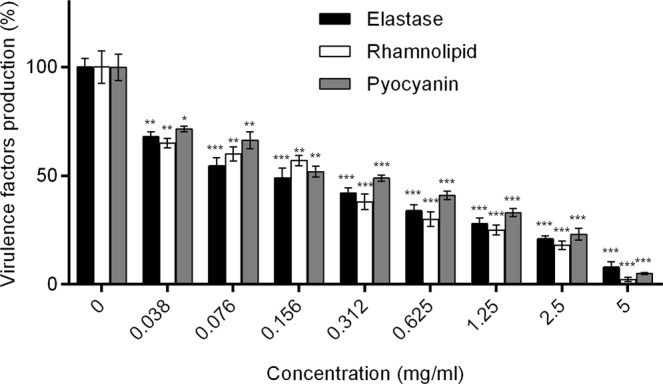
Graph demonstrating the dose-dependent effect of the *D. tsuruhatensis* 11304 QSI extract on *P. aeruginosa* MMA83 virulence factors production. Black bars – production of elastase, white bars – production of rhamnolipid and gray bars – production of pyocyanin. Statistical significance was evaluated by Student’s *t*- test against control (0) without QSI extract (*p < 0.05, **p < 0.01, ***p < 0.001).

The impact of the QSI extract on the QS regulated virulence factors (elastase, rhamnolipid and pyocyanin) production in *P. aeruginosa* MMA83 was next investigated. A dose-dependent decrease in the production of all the analyzed virulence factors was observed (Fig. 5). The lack of the production of elastase, rhamnolipid and pyocyanin after treatment of the *P. aeruginosa* MMA83 strain with a 5 mg/ml *D. tsuruhatensis* 11304 QSI extract has been noticed. The treatment with a 0.156 mg/ml QSI extract resulted in about 40% inhibition of rhamnolipid and 50% inhibition of elastase and pyocyanin production compared to the positive control.

### *D*. *tsuruhatensis* 11304 QSI extract inhibits the expression of *P*. *aeruginosa* MMA83 QS systems

**Figure 6:**
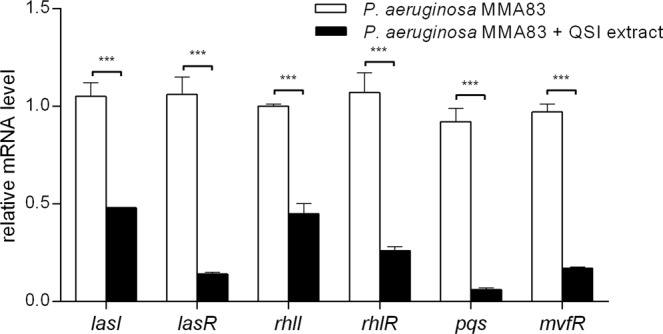
The changes of relative mRNA levels of QS genes without or with the treatment of *P. aeruginosa* MMA83 with a 5 mg/ml *D. tsuruhatensis* 11304 QSI extract. Genes examined in this study belong to three *P. aeruginosa* QS pathways (las – *lasI*, *lasR*; rhl – *rhlI*, *rhlR*; PQS – *pqs*, *mvfR*). RT-qPCR data were normalized against the ribosomal gene *rpsL* as an internal control. Student’s *t*- test was used to compare the differences between the control and experimental groups (***p < 0.001).

In order to confirm that the above described effects of *D. tsuruhatensis* 11304 QSI extract on *P. aeruginosa* MMA83 virulence were caused by transcriptional inhibition of QS systems genes belonging to three *P. aeruginosa* QS networks (las – *lasI*, *lasR*; rhl – *rhlI*, *rhlR*; PQS – *pqs*, *mvfR*) were selected for RT-qPCR study. The obtained results revealed that the treatment of *P. aeruginosa* MMA83 with a 5 mg/ml QSI extract significantly decreased the mRNA levels (***p < 0.001) (Fig. 6). Relative expression of the genes coding for inducer synthases *lasI* and *rhlI* was 2.2 times lower, while the expression of transcriptional regulators *lasR* and *rhlR* was even more reduced (7.6 and 4.1 times lower, respectively) compared to the control. The most significant downregulation of transcription was observed in the case of the *pqs* gene (15 times lower), while the transcription of transcriptional regulator *mvfR* was 5.7 times lower compared to the control.

### Determination of *D*. *tsuruhatensis* 11304 QSI extract composition by MALDI mass spectrometry

To define the nature of the molecules composing the *D. tsuruhatensis* 11304 QSI extract, an aliquot of the sample was preliminarily purified on a thin-layer chromatography (TLC) silica gel. Separated spots were visualized, and then scratched off. The silica gel was put into a cotton-plugged column, and the sample was eluted from the gel by ethyl acetate, dried and then investigated by MALDI mass spectrometry. Several solvents were employed to dilute and analyze the QSI extract; nevertheless, the best resolved spectra were obtained by diluting the QSI extract directly in the matrix solution (see Methods section for details).

**Figure 7:**
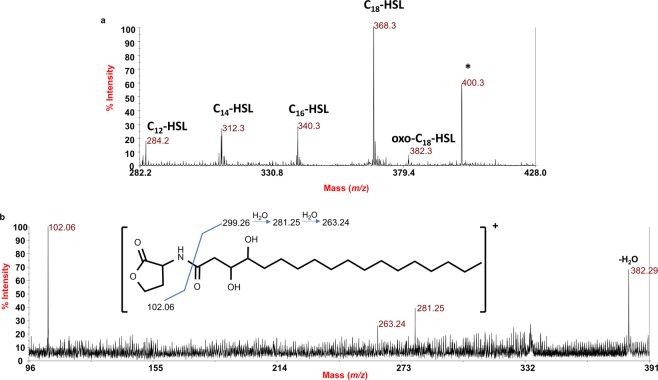
MALDI MS investigation of the *D. tsuruhatensis* 11304 QSI extract. (**a**) Positive ion MALDI MS spectrum recorded in reflectron mode of the *D. tsuruhatensis* 11304 QSI extract. (**b**) Positive ion MS^2^ spectrum of precursor ion at *m/z* 400.3. The relative AHL structure is reported in the inset. The position of the hydroxyl groups is tentative.

The positive ion MALDI mass spectrum, recorded in reflectron mode, is reported in Fig. 7a. The spectrum clearly indicated the presence of several [M + H]^+^ ions attributed to *N*-acyl homoserine lactones (AHLs) with chain lengths of C12 to C18. In particular, the main ion peak at *m/z* 368.3 was identified as *N*-octadecanoylhomoserine lactone (C_18_-HSL). This was proven by the observation in the related positive ion MS^2^ spectrum (Supplementary Fig. S3) of (**i)** the typical intense ion at *m/z* 102.06, common to all AHLs detected, relative to the protonated α-amino-γ-butyrolactone deriving from the cleavage of the carboxamide linkage between the acyl chain and the homoserine group; (**ii)** the counterpart acylium ion arising from the same cleavage (*m/z* 267.27) as well as (**iii)** the occurrence of a fragment originated from the loss of a water molecule from the [M + H]^+^ molecular ion (*m/z* 350.29)^32,33^. Similarly, other AHLs species with a shorter acyl chain have been identified at *m/z* 284.2 (C_12_-HSL), *m/z* 312.3 (C_14_-HSL) and *m/z* 340.3 (C_16_-HSL). In traces, *N*-3-oxo-octadecanoylhomoserine lactone (oxo-C_18_-HSL) has also been detected at *m/z* 382.3 (Fig. 7a). In addition, a clearly intense peak at *m/z* 400.3 was noted and isolated as MS^2^ precursor ion furnishing important structural data. The corresponding MALDI MS^2^ spectrum (Fig. 7b), revealed the occurrence of the common ion at *m/z* 102.06 but also of an ion derived from the loss of water from the protonated molecular ion (*m/z* 382.29).

### C_18_-HSL reduces pyocyanin production and quorum sensing *lasI* gene expression in *P. aeruginosa* MMA83

**Figure 8:**
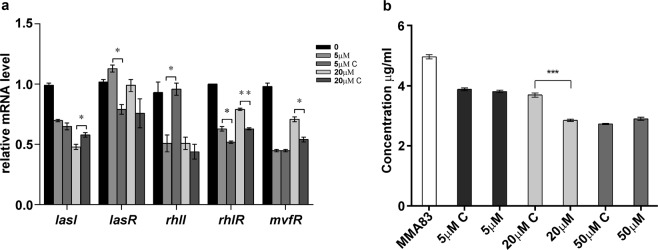
The QSI effect of commercial C_18_-HSL to *P. aeruginosa* MMA83. (**a**) The changes of relative mRNA levels of QS genes without or with the treatment of *P. aeruginosa* MMA83 with commercial C_18_-HSL. Genes examined in this study belong to three *P. aeruginosa* QS pathways (las – *lasI*, *lasR*; rhl – *rhlI*, *rhlR*; PQS – *mvfR*). RT-qPCR data were normalized against the ribosomal gene *rpsL* as an internal control. (**b**) The changes in pyocyanin production after the treatment of *P. aeruginosa* MMA83 with a commercial C_18_-HSL. “C” on both graphs refers to the DMSO control. Student’s *t*- test was used to compare the differences between the control and experimental groups (*p < 0.05, **p < 0.01, ***p < 0.001).

In order to investigate QSI potential of C_18_-HSL its effect on expression of quorum sensing genes and pyocyanin production in *P. aeruginosa* MMA83 was analyzed. Small but statistically significant decrease in pyocyanin production by MMA83 grown in presence of 20 µM C_18_-HSL was observed (Fig. 8b). In addition, the same concentration of C_18_-HSL statistically significant reduced transcription of the *lasI* gene in MMA83, although the fold-change was small (Fig. 8a).

## Discussion

Our understanding of *Delftia tsuruhatensis* has experienced a complete turnaround from the promising plant growth-promoting bacteria to emerging human pathogen predominantly isolated from respiratory specimens, blood and urine^27,30^. There is a disparity between the growing importance of *Delftia* spp. isolates and genomic data from these species. Noticeably, *D. tsuruhatensis* genomic data are scarce since only the genome of *D. tsuruhatensis* MTQ3 is publicly available on NCBI. Although we could not speculate about the origin of the *D. tsuruhatensis* 11304 strain, which was characterized in this study (was it intrahospital, or an environmental strain introduced to the hospital by a patient), the presence of genetic determinants for the production of siderophores, capsule and alginate is a good indication of a possible virulent phenotype. Along with the genetic determinants of antibiotic resistance that were found within the *D. tsuruhatensis* 11304 genome, like beta-lactamases and RND pumps, that could contribute to the overall pathogenicity of this particular strain and could provide insight into the genetic armory of *D. tsuruhatensis* in general.

The reason why we included *Delftia* spp. in testing was our initial presumption that bacteria, like *D. tsuruhatensis* 11304, which colonize the same tissues during infection of a host as *P. aeruginosa*, probably compete with *P. aeruginosa* by employing diverse mechanisms, including the interference of cell-to-cell signaling, i.e. quenching of the quorum sensing systems. Diisooctyl ester of 1,2 benzenedicarboxylic acid, a compound that is called by several different names, was previously identified as a QSI molecule, produced by *Delftia tsuruhatensis*, active against *P. aeruginosa* PAO1 QS^31^. It is worth noticing that a contamination of laboratory samples by this compound was found when plastic tubes were used, since it is widely used as a plasticizer^34,35^. In addition, our data showed that within the range of concentrations used in this study, bis (2-ethylhexyl) phthalate and diisooctyl phthalate were unable to interfere with *C. violaceum* CV026 quorum sensing system or with *P. aeruginosa* biofilm formation. Thus, our main aim was to characterize the QSI potential of *D. tsuruhatensis* 11304 against *P. aeruginosa* by using *C. violaceum* and a multidrug-resistant clinical isolate *P. aeruginosa* MMA83 as model systems. The *D. tsuruhatensis* 11304 ethyl acetate extract showed a remarkable ability to attenuate *P. aeruginosa* MMA83 QS, and to suppress its virulent phenotype by disabling its intrinsic ability to form a biofilm or to produce virulence factors such as elastase, rhamnolipid and pyocyanin.

Biofilm formation ability of *P. aeruginosa* MMA83 was significantly impaired by the *D. tsuruhatensis* 11304 extract, without affecting overall bacterial cell growth. This reduction in biofilm forming ability occurred in a dose-dependent manner, similarly as previously reported for other QSI extracts^36,37^. However, the disruption of preformed biofilm was not achieved, which might result from biofilm matrix impermeability or possibility that it acts through QS inhibition. The production of extracellular virulence factors is of crucial importance for the invasion of host tissues, initial phases of biofilm formation and promoting virulence expression and it was shown to be controlled by *las* and *rhl* QS systems^38,39^. Our study demonstrated that the *D. tsuruhatensis* 11304 extract was highly capable of attenuating *P. aeruginosa* virulence, and thus possibly infection, by significantly reducing the production of these virulence factors. This anti-virulent effect of the *D. tsuruhatensis* 11304 extract was based on the inhibition on transcriptional level of *las*, *rhl* and *pqs* QS systems, indicating a possible therapeutic potential of the QSI molecule(s) produced by strain 11304.

Considering that clinical isolates of *P. aeruginosa* often possess MDR or even XDR resistant phenotype, novel strategy for clinical treatment of infections caused by such isolates could be the combination of antibiotics with non-antibiotic bioactive compounds. It has been reported that different QS inhibitors caused a decrease in antibiotic resistance in *P. aeruginosa* PAO1 through synergistic effects with clinically used drugs^40,41^. In our study, susceptibility of the MDR clinical isolate *P. aeruginosa* MMA83 to meropenem and gentamycin was enhanced by synergistic interactions of the *D. tsuruhatensis* 11304 QSI extract with antibiotics.

MALDI MS data showed that AHLs with chain lengths of C12 to C18 were present in the *D. tsuruhatensis* 11304 QSI extract, with the main ion peak attributed to *N*-octadecanoylhomoserine lactone (C_18_-HSL). Interestingly, peak at *m/z* 400.3 was noted and isolated as MS^2^ precursor ion furnishing important structural data. The corresponding MALDI MS^2^ spectrum (Fig. 7b), revealed the occurrence of the common ion at *m/z* 102.06 but also of an ion derived from the loss of water from the protonated molecular ion (*m/z* 382.29). Two peaks at *m/z* 281.25 and 263.24, matching with the loss of one and two water molecules respectively from the acyl chain, suggested the occurrence of two hydroxyl groups decorating the acyl chain. None of the fragments helped in understanding the position of the two hydroxyl moieties; nevertheless, according to literature data, it could be assumed that a single hydroxyl group is placed at position 3 of the acyl chain whereas the position of the second remains to be defined. Therefore, a novel AHL species could be identified as composing the *D. tsuruhatensis* 11304 QSI extract, specifically a dihydroxy-*N*-octadecanoylhomoserine lactone (Fig. 7b). Although C_18_-HSLs were previously described^42^, to our best knowledge this is the first report of a naturally occurring dihydroxy-*N*-octadecanoylhomoserine lactone. Finding that commercial C_18_-HSL reduces *lasI* quorum sensing gene expression and pyocyanin production indicates its involvement in virulent potential of *P. aeruginosa* MMA83. This finding is of importance since it is known that, in certain bacterial species, long-chain AHLs interfere with the short-chain AHLs-mediated QS signaling^17,18^. For example, C_10_-HSL fails to activate the CviR-dependent transcription in *C. violaceum* CV026, and yet functions as an antagonist in the presence of a native autoinducer C_6_-HSL. Additionally, it was shown that the lengthening of the AHLs’ acyl-tails in the CV026 model system reduces agonism and enhances antagonism due to the promotion of an inactive conformation of the CviR:ligand complex^43^. It is tempting to speculate that the long-chain AHLs in the *D. tsuruhatensis* 11304 extract, among which a predominantly novel dihydroxy-*N*-octadecanoylhomoserine lactone is present, could be responsible for the interference of the *P. aeruginosa* MMA83 quorum sensing system and mitigation of its virulence. An analogy could be drawn to the CV026 quorum sensing system since *P. aeruginosa* doesn’t produce AHLs with side chains longer than C12^44^. Our findings could be also supported with observations made by Zhu *et al*.^45^, who showed that one of the strongest antagonists of TraR, which is a 3-oxo-C_8_-HSL-responsive transcriptional activator in *Agrobacterium tumefaciens*, had also hydroxyl residues placed at position 3 of the acyl chain. The authors of mentioned study also claimed that the length of the side acyl chain was one of key determinants affecting antagonistic properties of synthetic AHLs used in their model system. We should take into account that the observed QSI activity could not be attributed solely to C_18_-HSL but to an orchestrated activity of several molecules from the extract, due to limited effect of C_18_-HSL comparing to the effect of entire 11304 QSI extract. Thus, our further work will be focused on the separation and identification of all the components in *D. tsuruhatensis* 11304 ethyl acetate extract, and testing of their QSI activities independently.

## Methods

### Bacterial strains and cultivation conditions

Strain *Delftia* sp. 11304 from the collection of Laboratory for Molecular Microbiology (LMM), Institute of Molecular Genetics and Genetic Engineering (IMGGE), University of Belgrade, used in this study was isolated in a co-culture with *Achromobacter xylosoxidans* 11304 from a cough swab^46^ in a tertiary type hospital in Belgrade, Serbia. Quorum sensing inhibition (QSI) activity of 633 clinical isolates from LMM collection was tested by using AHL biosensor strain *Chromobacterium violaceum* 026 (CV026), a mini-Tn5 mutant deficient in the AHLs synthase *cvil*^17^. *Pseudomonas aeruginosa* MMA83, a New Delhi metallo-beta-lactamase-producing clinical strain from the laboratory collection (LMM, IMGGE) was used as a test strain^47,48^. The bacteria were cultured aerobically in either Luria–Bertani (LB) broth medium or Mueller-Hinton medium (MH) at 37 °C for all isolates except *C. violaceum* CV026 which was grown at 30 °C. M9 medium (10 x M9 salts - Na_2_HPO_4_x7H_2_O, KH_2_PO_4,_ NaCl, NH_4_Cl; 20% glucose; 1 M MgSO_4_; 1 M CaCl_2_; dissolved in miliQ water) supplemented with 0.24% pyruvate was used for bacterial cultivation on a large scale for extraction of molecule(s) that interfere with quorum sensing system.

### QSI detection assay

Initial screening for *Delftia* sp. 11304 QSI activity was performed with colorimetric agar well diffusion assay using *Chromobacterium violaceum* CV026 as an indicator strain. Overnight culture of *C. violaceum* CV026 (0.5% v/v) was inoculated in Luria-Bertani soft-agar (0.5% v/v) with addition of 5 *μ*M N-(hexanoyl)-l-homoserine lactone – HHL (Sigma-Aldrich, Missouri, USA) and overlaid on LB agar (17% v/v). Wells were made in lawn of the solidified LB soft agar. Wells were filled with 50 μl of *Delftia* sp. 11304 culture and incubated for 24 h at 30 °C in order to test QSI activity^49^. The absence of the violet color around the wells was considered as evidence of the inhibition of violacein pigment synthesis, and demonstrative of positive QSI activity.

QSI activity of *Delftia* sp. 11304 was monitored during different growth phases. Initially, 1% overnight culture was inoculated in fresh LB medium and grown at 37 °C, with aeration for 30 hours. Aliquots of bacterial culture were collected at different time points (0, 2, 4, 6, 8, 10, 12, 14, 16, 24 and 30 hours) and serial tenfold dilutions were prepared for determination of CFU/ml number. In parallel, QSI activity was determined by colorimetric agar well diffusion assay where diameter of QS inhibition was measured. Experiment was done in triplicate.

Chemical compounds bis (2-ethylhexyl) phthalate and diisooctyl phthalate that possess the same molecular formula C_24_H_38_O_4_ (molecular weight 390.564 g/mol) and the same structural formula (diisooctyl phthalate) as a previously described quorum sensing inhibitor compound^31^ were used in order to confirm their QSI and antibacterial activity. Two-fold serial dilutions (started from 1024 µg/ml to 4 µg/ml) of bis (2-ethylhexyl) phthalate and diisooctyl phthalate solutions (Sigma-Aldrich) dissolved in ethanol (1:1 ratio)-were tested for antibacterial activity on *C. violaceum* CV026 and *P. aeruginosa* MMA83, QSI activity (*C. violaceum* CV026) as well as biofilm forming effect (*P. aeruginosa* MMA83) using previously described methods.

### Whole genome sequencing and genome analyses

Genomic DNA of *Delftia* sp. 11304 was sequenced using Illumina HiSeq by MicrobesNG service (MicrobesNG, IMI-School of Biosciences, University of Birmingham, Birmingham, UK). The quality of each sequencing library was assessed using FastQC^50^. IDBA-UD with multi k-mer mode outperformed the assembly using De Bruijn Graph methods^51^. In addition, unassembled reads were collected and assembled by Celera Assembler with the Best Overlap Graph-CABOG. Finally, raw reads were mapped to assembled scaffolds with Burrows Wheeler Aligner- BWA^52^. *Delftia* sp. 11304 was identified using the genome sequence and EzBioCloud 16 S rRNA gene database available online at https://help.ezbiocloud.net/ezbiocloud-16s-database/^53^.

Identification of genes coding virulence factors in the genome sequence was performed using virulence factor database (VFDB), an online resource for curating information about virulence factors of bacterial pathogens (http://www.mgc.ac.cn/VFs/main.htm)^54^ and Linux command line. Presence of the antibiotic resistance genetic determinants in the sequenced genome was determined by the publicly available database, The Comprehensive Antibiotic Resistance Database (CARD, https://card.mcmaster.ca/)^55^ using Linux command line. Draft genome sequence of *Delftia tsuruhatensis* 11304 has been deposited at the NCBI GenBank database under accession number SMMJ00000000.

### Stability of *D. tsuruhatensis* 11304 QSI molecule(s)

In order to test the thermostability of *D. tsuruhatensis* 11304 QSI activity, overnight culture and cell-free supernatant were incubated at 100 °C for 30 and 60 min. Additionally, a potential proteinaceous nature of QSI activity was tested by treatment of *D. tsuruhatensis* 11304 overnight culture and cell-free supernatant with proteinase K (500 μg/ml) at 37 °C for 3 hours. Residual QSI activities after the heat or proteinase K treatments were evaluated by colorimetric agar well diffusion assay.

### Preparation of the *D. tsuruhatensis* 11304 QSI extract

Bacterial culture *D. tsuruhatensis* 11304 (five liters) was cultivated in M9 medium supplemented with 0.24% pyruvate for 24 h, at 37 °C, aerobically. After centrifugation at 13,680 x g, at 4 °C, for 30 min, the collected supernatant was divided into four glass flasks and each of them was extracted with an equal volume of methanol, chloroform, ethyl acetate and hexane (Sigma-Aldrich) with vigorous shaking for 30 min at room temperature. Extracts were evaporated to dry using a vacuum rotary evaporator at 50 °C for ethyl acetate and 30 °C for methanol, chloroform and hexane (Buchi Rotavapor, R200, Fisher Scientific, Hampton, New Hampshire, US) and the dry mass was dissolved in dimethyl sulfoxide (DMSO) for further analyses^56,57^. In order to avoid contamination with phthalates from plasticware all experiments were done in glassware.

### The effect of the *D. tsuruhatensis* 11304 QSI extract on *P. aeruginosa* MMA83 biofilm formation ability

In order to determine the minimal inhibitory concentration (MIC) of ethyl acetate QSI extract dissolved in DMSO on *P. aeruginosa* MMA83 planktonic cells, a 5 mg/ml QSI extract (following two-fold serial dilutions) was inoculated with 2 × 10^5^ CFU/ml of the test strain using a 96-wells microdilution method. The MIC value was defined as the lowest concentration which inhibited bacterial growth. Controls were incubated with a concentration of DMSO equal to that used in the treatment (0.5% v/v). Number of CFU/ml was determined by plating serial tenfold dilutions after 24 h of incubation at 37 °C. The experiments were performed in sextuplicate and repeated three times. Afterwards, wells were washed to remove planktonic bacterial cells and stained with 0.1% (v/v) crystal violet (HiMedia Labs Pvt. Ltd., India)^58^. Biofilm formation was quantified by recording the absorbance at 595 nm using Plate Reader Infinite 200 pro (MTX Lab Systems, Austria).

The effects of the QSI extract on biofilm formation or decomposition of preformed biofilm were additionally visualized using fluorescent dye SYTO9 (TermoFisher Scientific, Massachusetts, USA) and propidium iodide (PI) (Sigma-Aldrich)^59^. For visualization of biofilms, *P. aeruginosa* MMA83 (2 × 10^5^ CFU/ml) was cocultivated with a 5 mg/ml QSI extract in 24-well plates (Tissue Culture Plate, Sarstedt, Germany) which contained microscopic cover glass. After incubation (24 h, at 37 °C), the cells were washed 3 times with PBS and stained with SYTO9 (2.5 µM) and PI (2.5 µM), green and red fluorescent dyes, respectively. In order to test the decomposition of preformed biofilm in the presence of the QSI extract, *P. aeruginosa* MMA83 was cultivated for 24 hours, at 37 °C in 24-well plates, washed 3 times with PBS and, subsequently, treated with 5 mg/ml of QSI extract. After next 24 hours (48 hours in total), cells were washed and stained as previously described. Stained cells were visualized by fluorescence microscope (Olympus BX51, Applied Imaging Corp., San Jose, California, USA) under 20,000 × magnification. Untreated bacterial cells were used as a positive control.

### QSI extract effect on antibiotic MIC values against *P. aeruginosa* MMA83

The combined effect of the *D. tsuruhatensis* 11304 QSI extract and selected, clinically relevant antibiotics against *P. aeruginosa* MMA83 were assessed using 96-well plate microdilution method. Serial dilutions of meropenem (2.048, 1.024, 0.512, 0.256, 0.128, 0.064, 0.032, 0.016, 0.008, 0.004 or 0.002 mg/ml) or gentamycin (4.096, 2.048, 1.024, 0.512, 0.256, 0.128, 0.064, 0.032, 0.016, 0.008, 0.004 or 0.002 mg/ml) were cross-diluted with serial dilutions of QSI extract (20, 10, 5, 2.5, 1.25, 0.625, 0.312, 0.156, 0.076 or 0.038 mg/ml) and DMSO (16, 8, 4, 2, 1, 0.5%). Different concentrations of DMSO were expressed in mg/ml (16% corresponds with 145.45 mg/ml, etc.) in order to calculate final outcome for fractional inhibitory concentration. Plates (Tissue Culture Plate, Sarstedt, Germany) were filled with the bacterial suspension at a final density of 2 × 10^5^ CFU/ml. Cell density was recorded by OD_600_ measurements using Plate Reader Infinite 200 pro (MTX Lab Systems, Austria) after 24 hours of incubation at 37 °C. The experiments were performed in triplicate and repeated two times.

The fractional inhibitory concentrations (FICs) were determined according to the previously described checkerboard method^60^.The outcome was defined as a synergistic if the sum of two FICs (FIC of the antibiotic and FIC of the QQ extract) was ≤ 0.5; additive if 0.5 < ∑FIC ≤ 1; indifferent if 1* < *∑FIC < 4; antagonistic if ∑FIC > 4.

### The effect of the *D. tsuruhatensis* 11304 QSI extract on the virulence factors production in *P. aeruginosa* MMA83

*Elastase assay*. Supernatants of overnight culture of *Pseudomonas aeruginosa* MMA83 were supplemented with two-fold diluted concentrations of the QSI extract (starting with 5 mg/ml, down to 0.038 mg/ml) and then mixed with Elastin-Congo red (Sigma-Aldrich) at a final concentration of 2 mg/ml. After 24 h of incubation at 37 °C, with shaking (180 rpm), the mixtures were centrifuged at 15,700 x g for 15 min, after which the elastase activity was measured at 495 nm using Plate Reader Infinite 200 pro (MTX Lab Systems, Austria)^61^. Supernatant of *P. aeruginosa* MMA83 culture without the QSI extract treatment was used as a positive control.

*Rhamnolipid assay*. Rhamnolipid production was examined by acidification of supernatants with HCl (to pH 2) of the previously cultivated test strain (co-incubated with two-fold serial dilutions of the QSI extract or without the QSI extract)^62^. The absorbance was monitored spectrofotometrically at 570 nm by Plate Reader Infinite 200 pro (MTX Lab Systems, Austria).

*Pyocyanin assay. P. aeruginosa* MMA83 overnight culture (with two-fold dilutions of the QSI extract or without the QSI extract) was centrifuged at 15,700 x g for 15 min and pyocyanin was extracted from the supernatant using chloroform in a 1:2 ratio following the re-extraction of chloroform phase by 0.2 N HCl (3:1 ratio) according to the previously described method^63,64^. The concentration of pyocyanin was evaluated by measuring the absorbance of the red top layer at 520 nm using Plate Reader Infinite 200 pro (MTX Lab Systems, Austria). Concentrations, expressed as micrograms of pyocyanin produced per milliliter of culture supernatant, were determined by multiplying the optical density at 520 nm (OD_520_) by 17.072.

### Quantification of *P. aeruginosa* MMA83 quorum sensing genes expression by RT-qPCR

Reverse transcription-quantitative PCR (RT-qPCR) was performed to determine the expression levels of regulatory genes involved in quorum sensing. Primers used for the amplification of selected genes are listed in the Table 2. The total RNA was isolated from the test strain *P. aeruginosa* MMA83 (grown for 10 hours, at 37 °C) supplemented with the QSI extract (5 mg/ml) or without it by RNeasy Mini Kit (Qiagen, Germany). The total RNA was then treated with DNase using an Ambion DNA-free^TM^ Kit (ThermoFisher, MA, US) and reverse transcribed by a Rever-tAid RT Reverse transcription Kit (ThermoFisher) according to the manufacturer’s instructions. Further amplification was achieved with KAPA SYBR Fast qPCR Kit (Kapa Biosystems, Wilmington, MA, USA) in a 7500 Real Time PCR System (Applied Biosystems, Waltham, MA, USA) under the following cycling conditions: incubation at 95 °C for 3 min and 40 cycles of 95 °C/15 s and 60 °C/1 min. RT-qPCR data was normalized against the ribosomal gene *rpsL* as an internal control following the 2^-ΔΔCt^method^65^.

**Table 2:** List of primers used for RT-qPCR.

Gene	Primer direction	Sequence (5′-3′)	Amplicon size (bp)	Source
*lasI*	Forward	5-GCGTGCTCAAGTGTTCAAGG-3	125	This study
	Reverse	5-GGGCTTCAGGAGTATCTTCCTGG-3		This study
*lasR*	Forward	5-GGAGTGGAGCGCCATCCTGCAG-3	127	This study
	Reverse	5-GGCGGCCGGGTAGTTGCCGACG-3		This study
*rhlI*	Forward	5-CCATCCGCAAACCCGCTACATC-3	151	This study
	Reverse	5-CTCCCAGACCGACGGATCGCTCGGC-3		This study
*rhlR*	Forward	5-GGGCGTGTTCGCCGTCCTGG-3	143	This study
	Reverse	5-GGTATCGCTCCAGCCAGGCCTTG-3		This study
*pqsA*	Forward	5-CCGGACCTACATTCTCTCCC-3	182	This study
	Reverse	5-CGATATCGGCCAGGGCCTGC-3		This study
*mvfR*	Forward	5-GTCGGGACGGCTACAAGGTCG-3	129	This study
	Reverse	5-GATTGCGCGGACCCTTGTTGAG-3		This study
*rpsL*	Forward	5-GCAACTATCAACCAGCTGGTG-3	231	This study
	Reverse	5-GCTGTGCTCTTGCAGGTTGTG-3		This study

### Isolation of QSI molecules by TLC

An aliquot (1 mg) of the *D. tsuruhatensis* 11304 QSI extract was dissolved in 100 µl of ethyl acetate and was applied to TLC (Silica gel 60, F_254_, Aluminium sheets, Merck, Darmstadt, Germany) followed by developing with a mixture of chloroform and ethyl acetate in the ratio 5:4.

Separated spots were scratched off from the plate and the silica gel was put into a cotton-plugged column. The sample was then eluted from the silica gel with ethyl acetate.

### MALDI MS and MS^2^ of the *D. tsuruhatensis* 11304 QSI extract

The MS structural analysis was performed on an ABSCIEX TOF/TOF^TM^ 5800 Applied Biosystems mass spectrometer equipped with an Nd:YLF laser (λ = 345 nm), with a pulse length of <500 ps and a repetition rate of up to 1000 Hz. The dried sample obtained after TLC separation was redissolved either in acetonitrile, ethyl acetate, or directly in an α-cyano-4-hydroxycinnamic acid matrix solution (5 mg/ml) in acetonitrile/TFA 0.1% (70:30)^32^. The prepared QSI extract (0.5 µl) and matrix solution (0.5 µl), or 1 µl of the mixture were deposited on a stainless-steel plate and left to dry at room temperature. Each spectrum, acquired in positive ion mode, was a result of the accumulation of 2,000 laser shots, whereas 2,500 shots were summed for the MS^2^ data acquisitions.

### The effect of the commercial C_18_-HSL on the pyocyanin production and quantification of quorum sensing genes expression in *P. aeruginosa* MMA83

Commercial *N*-octadecanoylhomoserine lactone (C_18_-HSL) was purchased from Cayman Chemical (Tallin, Estonia) and resuspended in DMSO (0.5 mg/ml) according to manufacturer instructions. Concentrations of 5, 20 and 50 µM C_18_-HSL were used for evaluation of pyocyanin production and quantification of quorum sensing genes (listed in Table 2) expression in the presence of C_18_-HSL. Each concentration of C_18_-HSL was compared with control containing the exact volume of DMSO without C_18_-HSL to eliminate biological effect of DMSO in the experiment. Pyocyanin productions was expressed in µg/ml as described previously^63,64^. Experiments were done as described above, in triplicate.

### Statistical analyses

The statistical analyses and visualization were performed using GraphPad Prism software and SPSS 20.0 for Windows. The results are shown as means ± standard errors. The differences between control and experimental groups were compared using Student’s *t-*test. A *p* value less than 0.05 was considered to be statistically significant.

## Supplementary information

Dataset 1

Dataset 2

Dataset 3
