# Supplementary material for: Pseudomonas aeruginosa quorum sensing inhibition by clinical isolate Delftia tsuruhatensis 11304: involvement of N-octadecanoylhomoserine lactones
Source: Sci Rep. 2019 Nov 11;9:16465. doi: 10.1038/s41598-019-52955-3 (PMC6848482; doi:10.1038/s41598-019-52955-3)
Supplement: Supplementary file 1 — Dataset 1 [file 41598_2019_52955_MOESM1_ESM.docx]

**Supplementary data**

***Pseudomonas* *aeruginosa* quorum sensing inhibition by clinical isolate *Delftia tsuruhatensis* 11304: involvement of *N*-octadecanoylhomoserine lactones**

Milka Malešević^1^, Flaviana Di Lorenzo^2^, Brankica Filipić^1, 3^, Nemanja Stanisavljević^1^, Katarina Novović^1^, Lidija Senerovic^1^, Natalija Polović^4^, Antonio Molinaro^2^, Milan Kojić^1^, Branko Jovčić^1, 5^

^1^Institute of Molecular Genetics and Genetic Engineering, University of Belgrade, Belgrade, 11010, Serbia ^2^University of Napoli Federico II, Department of Chemical Sciences, Napoli, 80126, Italy ^3^Faculty of Pharmacy, University of Belgrade, Belgrade, 11221, Serbia ^4^Faculty of Chemistry, University of Belgrade, Belgrade, 11000, Serbia ^5^Faculty of Biology, University of Belgrade, Belgrade, 11000, Serbia

*Correspondence: Branko Jovčić bjovcic@bio.bg.ac.rs

**Supplementary table S1.** The 633 clinical isolates belonging to 11 different bacterial taxa from Laboratory for Molecular Microbiology, Institute of Molecular Genetics and Genetic Engineering, University of Belgrade collection, analyzed in this study.

| **Bacterial taxon** | **Number of tested isolates** |
| --- | --- |
| *Acinetobacter* spp. | 39 |
| *Achromobacter* spp. | 128 |
| *Burkholderia cepacia* complex | 218 |
| *Delftia* spp. | 2 |
| *Klebsiella* spp. | 27 |
| *Escherichia coli* | 1 |
| *Chryseobacterium* spp. | 38 |
| *Ochrobactrum* spp. | 5 |
| *Ralstonia* spp. | 26 |
| *Pseudomonas* spp. | 2 |
| *Stenotrophomonas* spp. | 147 |

**Supplementary table S2.** The 19 (belonging to five genera) of 633 analyzed strains that showed quorum sensing inhibitory activity according to colorimetric agar well diffusion assay. Diameter of inhibition zones of violacein production by *C. violaceum* CV026 was detrmined for each strain.

| **Bacterial genera** | **Designation of the strain** | **Zone diameter (mm)** |
| --- | --- | --- |
| *Achromobacter* spp. | 13011 | 14 |
| *Burkholderia cepacia* complex | BCC7  BCC8  BCC9  BCC19  BCC20  BCC24  BCC66  BCC301  BCC302  BCC532  BCC4135 | 17  16  17  12  13  15  17  17  19  19  20 |
| *Delftia* spp. | 11304  6960/2 | 23  17 |
| *Stenotrophomonas* spp. | 1556  6646  6960  7451 | 11  12  15  14 |
| *Ralstonia* spp. | 630 | 13 |

**Supplementary table S3.** MIC value of bis (2-ethylhexyl) phthalate and diisooctyl phthalate against test strains *C. violaceum* CV026 and *P. aeruginosa* MMA83.

| Name of the bacterial strain | MIC (µg/ml) | |
| --- | --- | --- |
|  | bis (2-ethylhexyl) phthalate | diisooctyl phthalate |
| *C. violaceum* CV026 | >1024 | >1024 |
| *P. aeruginosa* MMA83 | >1024 | >1024 |

**Supplementary table S4.** List of virulence factors genes found in *D. tsuruhatensis* 11304 genome using virulence factor database (VFDB) and Linux command line.

**Supplementary table S5.** List of antibiotic resistance determinants detected in *D. tsuruhatensis* 11304 genome**.** CARD database was used for the prediction of the *D. tsuruhatensis* 11304 resistome**.**


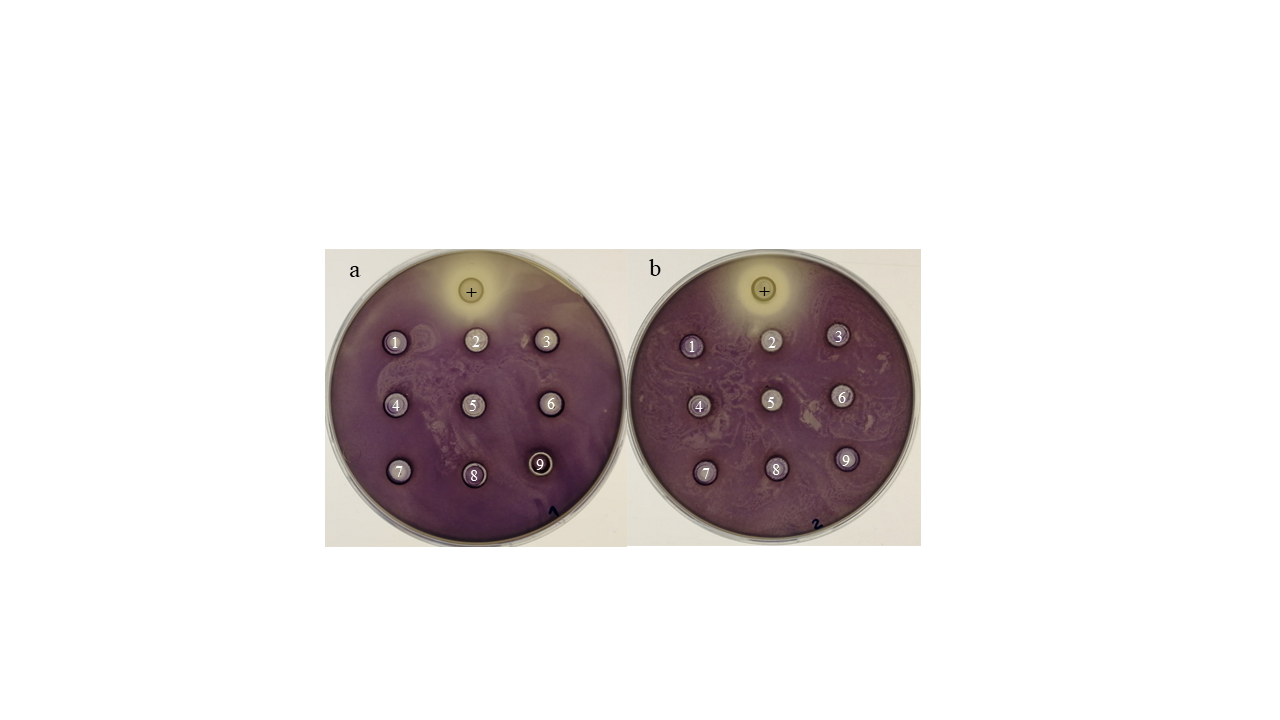


**Supplementary figure S1.** The effect of different concentrations (4 µg/ml to 1024 µg/ml) of **(a)** bis (2-ethylhexyl) phthalate and **(b)** diisooctyl phthalate on violacein pigment production. Neither of these compounds showed QSI activity at the used concentrations when tested with a colorimetric agar well diffusionassay using *Chromobacterium violaceum* CV026 as an indicator strain. Different concentrations are labeled from 1-9 following the order: 1-1024 µg/ml; 2-512µg/ml; 3-256µg/ml; 4-128µg/ml; 5-64µg/ml; 6-32µg/ml; 7-16µg/ml; 8-8µg/ml; 9-4µg/ml. Positive control (*D. tsuruhatensis* 11304 culture) is designated with ’+’.


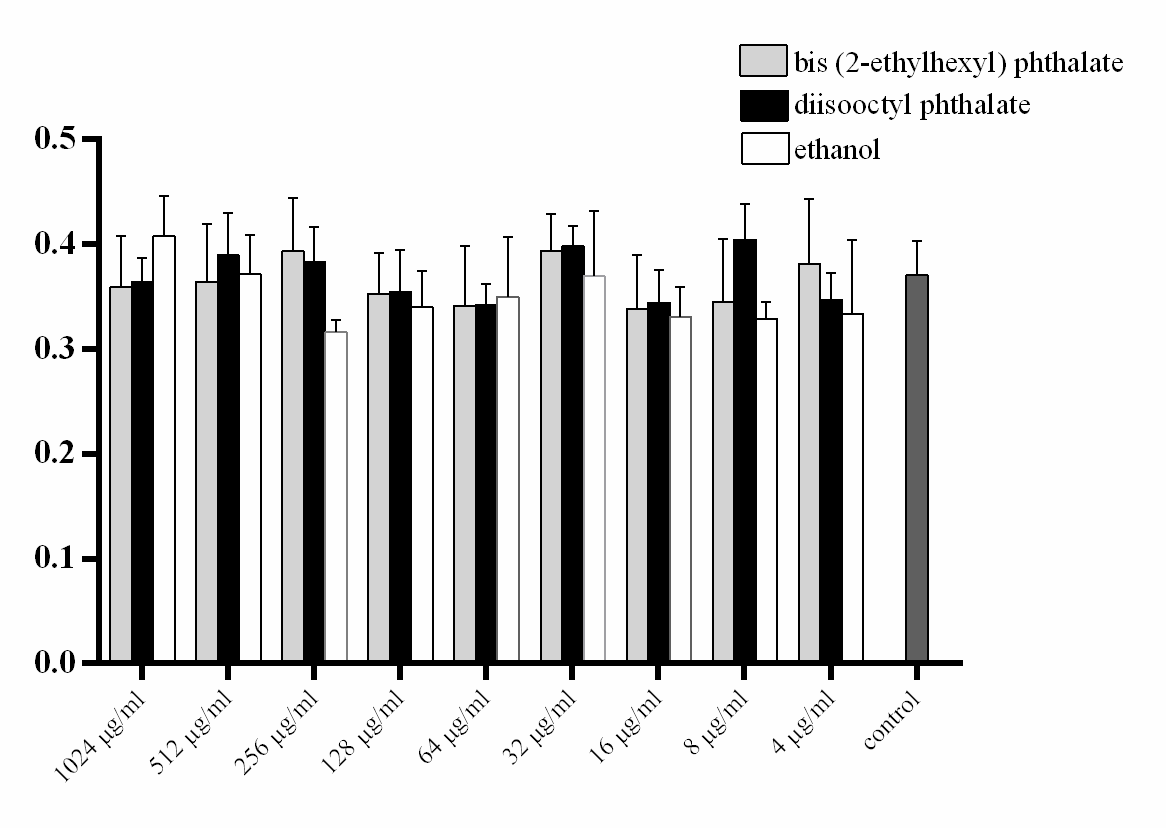


**Supplementary figure S2.** The effect of different concentrations of bis (2-ethylhexyl) phtalata and diisooctyl phtalate on biofilm formation of *Pseudomonas aeruginosa* MMA83. Axis – optical density of at 600 nm.


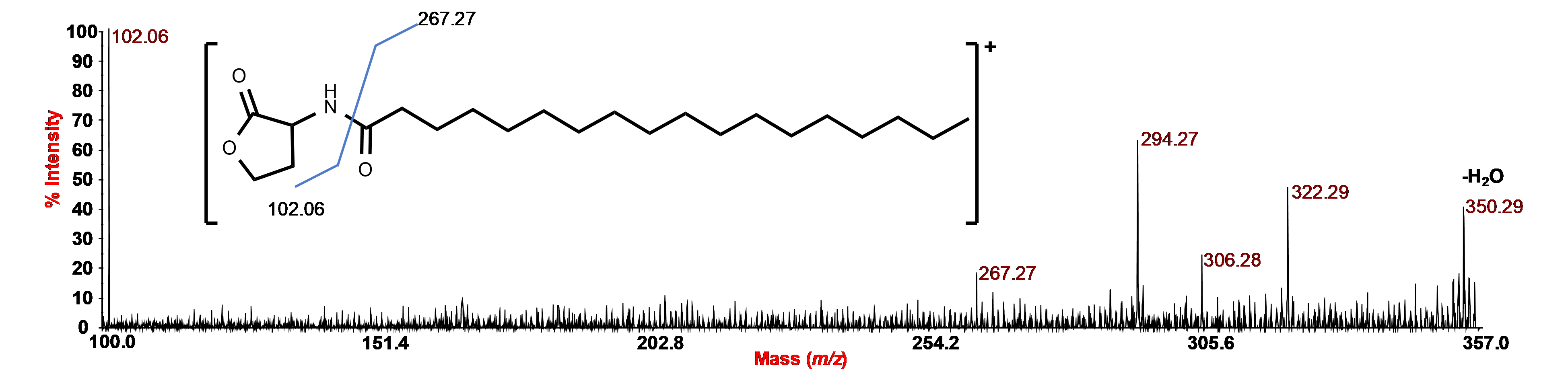


**Supplementary figure S3.** MALDI MS^2^studyof *N*-octadecanoylhomoserine lactone (C_18_-HSL). Positive ion MS^2^ spectrum of precursor ion (C_18_-HSL) is at *m/z* 368.3. The relative C_18_-HSL structure is reported in the inset.
